# Maternal-neonatal listeriosis

**DOI:** 10.1080/21505594.2020.1759287

**Published:** 2020-05-03

**Authors:** Caroline Charlier, Olivier Disson, Marc Lecuit

**Affiliations:** a Institut Pasteur, Biology of Infection Unit, Paris, France; b Inserm U1117, Paris, France; c Institut Pasteur, French National Reference Center and WHO Collaborating Center for Listeria, Paris, France; d Hôpital Universitaire Necker-Enfants Malades, Service des Maladies Infectieuses et Tropicales, Institut Imagine, APHP, Paris, France; e Université de Paris, Paris, France

**Keywords:** Listeriosis, *Listeria monocytogenes*, placenta, pregnancy, newborn, fetus, infection

## Abstract

Listeriosis is a rare and severe foodborne infection caused by *Listeria monocytogenes*. It manifests as septicemia, neurolisteriosis, and maternal-fetal infection. In pregnancy, it may cause maternal fever, premature delivery, fetal loss, neonatal systemic and central nervous system infections. Maternal listeriosis is mostly reported during the 2nd and 3rd trimester of pregnancy, as sporadic cases or in the context of outbreaks. Strains belonging to clonal complexes 1, 4 and 6, referred to as hypervirulent, are the most associated to maternal-neonatal infections. Here we review the clinical, pathophysiological, and microbiological features of maternal-neonatal listeriosis.

## Introduction

*Listeria monocytogenes* is the Gram-positive facultative intracellular bacillus responsible for listeriosis, a rare but particularly severe foodborne infection. Listeriosis can present as a self-limited gastroenteritis in immunocompetent individuals, as bacteremia and central nervous system infection (neurolisteriosis) mainly in immunocompromised individuals, the elderly, and maternal-neonatal (MN) listeriosis in pregnant women.

As evidenced by recent outbreaks, including that of South Africa 1,2, maternal-neonatal listeriosis remains one of the infections associated with the highest fetal and neonatal morbidity, leading to fetal losses in at least 25% of cases, and to severe neonatal infections or prematurity in most other cases [3–5]. After ingestion, *L. monocytogenes* can actively cross the intestinal barrier, disseminate via the bloodstream, and eventually cross the placental barrier, leading to placental and fetal infection [6,7]. A better understanding of the events leading to fetal and neonatal infection is critical to better prevent and manage MN listeriosis. Major advances in this regard have been made over the two last decades. Here, we briefly review the epidemiology and clinical features of MN listeriosis and present the current understanding of the pathophysiology of MN listeriosis.

## Clinical and microbiological epidemiology

*Surveillance –* The identification in the nineteen-eighties of *L. monocytogenes* as a major foodborne pathogen led to the implementation of a surveillance system in most countries of the Western hemisphere [8]. Altogether, epidemiological surveillance based on voluntary or mandatory reporting, microbiological surveillance relying on National reference laboratories and efficient food control procedures led to a sharp decrease of listeriosis cases, in particular in pregnant women [9,10]. The incidence of MN listeriosis, defined by the presence of *Listeria* in any sample of maternal, fetal or neonatal origin (infant 1-month old or younger), is now estimated around 4-10/100,000 pregnant women/year in Europe and North America [11,12]. MN listeriosis cases are more frequent in countries where surveillance is not thoroughly implemented [2,13,14]. Maternal-neonatal infections account for 11-20% of hospitalizations for invasive listeriosis in France and Spain [5,13]. Maternal infections may occur as sporadic cases or in the context of outbreaks, in particular in countries where food safety regulation or surveillance of listeriosis are not implemented. This is illustrated by the recent outbreak in South Africa, which started in late 2017 and is so far the largest reported worldwide, involving around 1,000 cases, including 42%-infected fetuses and neonates [2,15,16]. Most cases are reported during pregnancy second and third trimester; this could in part reflect a reporting bias, as *Listeria*-associated early fetal losses are likely neither diagnosed nor reported [5,17,18].

*Pregnancy is a major risk factor for listeriosis*, with a crude incidence estimated as 10 to 100 times higher in this group than in the general population [17–20]. Among pregnant women, higher incidences have been reported in ethnic minorities, probably reflecting specific dietary habits, such as for American Hispanic women in the US [19], women of African origin in France [5], and in women from ethnic minorities in the United Kingdom [21,22]. They may not be as aware as others of the preventive measures and may not have readily access to medical care in case of fever or obstetrical signs.

*Food sources – L. monocytogenes* is ubiquitous in nature [23] and can contaminate a large array of unprocessed and processed food of animal and vegetal origin. Most outbreaks have involved unpasteurized dairy products, but also meat-derived products and ready-to-eat food. Other food vehicles have also been reported, such as caramel apples or soybean sprouts[1].

*Genomic characterization of MN-associated strains – L. monocytogenes* may be isolated from clinical, animal, food, or environmental samples. It has been classified in lineages and clonal complexes (CCs) based on MLST (Multi-Locus Sequence Typing) and sublineages and cgMLST types based on cgMLST (core genome MLST) [24,25]. CC1, CC2, CC4, and CC6, which belong to lineage 1 and serotype 4b, are over-represented in MN infections isolates in France, with more than 2/3 of cases due to one of these CCs. These strains are also overrepresented in dairy products [26]. Among them, CC4 is the most associated with MN infections, 20% of CC4 isolates being of MN origin [27]. In contrast, CC9 and CC121, which belong to lineage 2, are the most frequent clonal complexes isolated from food but are almost never associated with MN infection [5,27]. This uneven distribution correlates with virulence and *L. monocytogenes* adaptation to mammalian gut: CC1, CC4 and CC6 colonize better the intestinal lumen and are hypervirulent, whereas CC9 and CC121 are hypovirulent in a mouse model of listeriosis, in part because they mostly express a truncated and nonfunctional form of InlA (see below) [26,27]. Moreover, CC4 displays a greater tropism for the placenta upon intravenous inoculation than reference strains historically used in research (EGDe from CC9 and **10403S** from CC7) [27]. This suggests a contribution of CC4-specific genes in placental infection (see below) [27]. Distribution of CCs and sublineages may differ according to countries. In China, ST87 is the most frequent MLST type associated with clinical cases, especially in pregnancy-associated cases [28,29]. Of note, this sequence type, which has been associated with human cases in Spain [30] contains the LIPI-4 gene cluster, as does CC4 (see below) [31].

## Clinical features

*The incubation period of MN* has been estimated from clinical cases where a contaminating food source had been identified. MN listeriosis is associated with a median incubation time of 19 to 27.5 days according to available studies (range: 7–67 days), longer than for neurolisteriosis (9 days; range: 1–14 days) and bacteremia (2 days; range: 1–12 days) [32,33]. This may reflect the time needed for maternal bacteremia, placental, and fetal infection to develop.

*Maternal presentation* falls into two main patterns, namely nonspecific obstetrical signs (uterine contractions, labor or abnormal fetal heart rate) and fetal loss [3,5,18], respectively, reported in 75% and 21% in the prospective MONALISA cohort from France [5]. It should be noted that fever is not always present (reported in 12% to 85% [3,5,18]), explaining why the diagnosis of MN listeriosis can be challenging. Maternal central nervous system involvement is absent in otherwise healthy pregnant women. Indeed, it has only been exceptionally reported [34], in pregnant women with immunosuppressive comorbidities [16]. Pregnancy is therefore not a risk factor *per se* of maternal neurolisteriosis, and CSF assessment should not be performed in otherwise non-immunosuppressed pregnant women with MN listeriosis. Placental sample culture is the most sensitive method to diagnose MN listeriosis, and should be performed together with maternal blood cultures, which are positive in 80% and 55% of the MN cases of the MONALISA cohort, respectively [5]. Maternal vaginal samples are usually negative, reflecting the hematogenous seeding of the placenta [5].

*Obstetrical outcome* ranges among the worst among MN infections. Upon maternal listeriosis, only 5% of pregnant women experience uneventful subsequent pregnancy and delivery [5]. Data from the MONALISA cohort evidenced major complications in 82% of pregnant mothers (88/107), including fetal losses (25% 27/107), premature deliveries before 32 weeks of gestation (19% of the maternal cohort (18/107), and 42% of all prematurely born children), and birth of infants with early or late onset listeriosis in the remaining cases [5]. Importantly, despite recommendations for ampicillin-based preemptive therapy for maternal fever in France, the rate of fetal losses related to listeriosis has not decreased over the last decades [3–5,18].

## Pathophysiology

*Placental tropism of L. monocytogenes –* In contrast to neurolisteriosis and *L. monocytogenes* bacteremia, MN listeriosis is not associated with immunosuppression other than pregnancy itself [5]. Moreover, apart from nonspecific obstetrical signs, fever or flu-like symptoms are the only clinical signs in infected mothers, and maternal mortality rate is 0%, compared to 30% and 46% for neurolisteriosis and bacteremia, respectively, [5]. Altogether, this suggests that *L. monocytogenes* exhibits a specific placental tropism.

*Experimental models of MN listeriosis* – *In vitro, ex vivo* and *in vivo* models have been instrumental to study experimentally *L. monocytogenes* placental infection. Cytotrophoblasts are placental epithelial cells of fetal origin that constitute the barrier between the maternal blood and the fetus. They fuse to generate the syncytiotrophoblast, which is in direct contact with maternal blood in the villous human placenta. Human trophoblast cell lines that can fuse in syncytiotrophoblasts, such as BeWo and Jar, allow studying *L. monocytogenes* interaction and invasion of cells that constitute the placental barrier. Human placental explants of first and third trimester are also instrumental to study *L. monocytogenes* crossing of the placenta barrier in a tissue context. *L. monocytogenes* can infect extravillous trophoblasts (EVT) [35] and syncytiotrophoblast (SYN) [36] in these experimental systems. Immunohistochemical analyses have demonstrated that *L. monocytogenes* infects SYN of placentas obtained from women with MN listeriosis [36]. Most of the reported cases of MN listeriosis occur late during pregnancy, suggesting that SYN is likely a major entry point for *L. monocytogenes* in the placenta [5]. It should be noted however that the occurrence of first trimester MN listeriosis is likely underestimated since miscarriage products at this gestational age are usually not available for microbiological investigations. This together with the fact that the placenta actively invades the uterine wall during the  first pregnancy trimester could account for a different placenta entry site of *L. monocytogenes*, with EVT being a target of *L. monocytogenes* early in pregnancy.

Even though placental explants have proven very useful to study placental infections [37], *in vivo* models are irreplaceable to fully decipher the pathophysiology of MN listeriosis. *L. monocytogenes* entry in host cells is species-specific. Indeed, two proteins of *L. monocytogenes*, InlA (internalin) and InlB, are critically involved in *L. monocytogenes* invasion of cultured epithelial cells and have host-specific receptors. InlA interacts with the *adherens* junction protein human E-cadherin (hEcad), but not with its murine ortholog (mEcad). This species specificity relies on the nature of a single amino acid located in position 16^th^ of mature Ecad. In permissive species (human, guinea pig, and gerbil), it is a proline, whereas it is a glutamic acid in non-permissive murine species (mouse and rat) [38]. Similarly, InlB interacts with human, mouse, and gerbil hepatocyte growth factor receptor c-Met, but not guinea pig and rabbit c-Met [39]. Non-human primates, especially pregnant Old-world monkeys including cynomolgus macaques (*Macaca fascicularis)* and rhesus monkeys (*Macaca mulatta*), are relevant models to study human listeriosis, since they harbor a villous hemochorial placenta like humans, in contrast to the labyrinthine placenta of rodents [40,41]. In Old-world monkeys, *L. monocytogenes* infection has been shown to induce stillbirth during the third [42,43] and first trimester [44]. However, ethical and cost limitations restrict the use of non-human primates for experimental purposes. Gerbil is a natural host for *L. monocytogenes* [45], and, as human, is permissive to both InlA-Ecad and InlB-Met interactions [6,46]. However, there are little genetic and analytical tools available in this species, which is also outbred and therefore exhibits significant inter-individual genetic variability. To circumvent these limitations, a knock-in E16P (KIE16P) mouse line has been generated [6], where the glutamic acid in position 16 of mouse Ecad (mEcad) is replaced by a proline. This substitution creates a so-called humanized Ecad enabled to interact with InlA where it is expressed *in vivo* [6]. KIE16P mice constitute therefore a model of choice to study the pathophysiology of human listeriosis, including maternal-fetal infection.

*Dynamic of L. monocytogenes placental infection* – Entry in placenta tends to be clonal in experimentally infected animals, as shown by the distribution of competitive indexes in gerbils and KIE16P mice [6,27]. However, once the placenta is infected, bacterial load increases rapidly, even in InlB non-permissive animals such as the guinea pig (cf. infra) [47]. In this species, bacteria shed back from the placenta to maternal organs, indicating that the placenta can behave as a reservoir that can seed in return maternal organs thereby enhancing within-host bacterial load [47].

**Figure 1.**
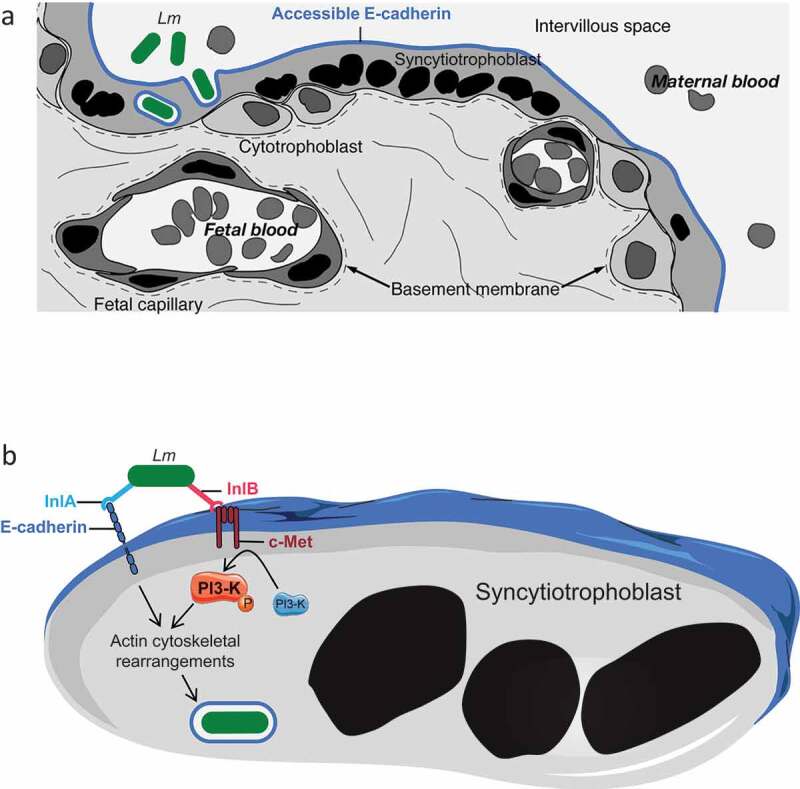
InlA and InlB dependent breaching of the placental barrier by *Listeria monocytogenes.* (a) The placental barrier between the maternal blood and the fetus lies in an epithelium, the syncytiotrophoblast, which results from the fusion of underlying cytotrophoblast cells. Syncytiotrophoblast expresses E-cadherin, which is accessible for bacteria in the maternal blood. (b) *Listeria monocytogenes* adheres to syncytiotrophoblast via InlA interaction with Ecad. InlB is required for *L. monocytogenes* entry by activating PI3-K via c-Met in the syncytiotrophoblast, leading to the actin cytoskeleton rearrangements needed for bacterial internalization.

*Mechanism of placental barrier crossing: the critical role of InlA and InlB –* The study of histological sections of placentas from actual cases of human MN listeriosis revealed the presence of *L. monocytogenes* in the SYN cytoplasm and villus stroma [36]. Because both Ecad and c-Met are expressed on the surface of SYNs and EVT, the role of InlA and InlB in placental barrier invasion has been investigated in detail (Figure 1) [36]. The potential role of InlA was first highlighted epidemiologically, since 100% of the clinical strains responsible for MN listeriosis express a non-truncated InlA, a significantly higher proportion than strains isolated from cases of bacteremia (93%), and isolated from food (65%) [5,27,48]. InlA is involved in the attachment to and invasion of human BeWo (SYN-differentiated) and Jar trophoblastic cell lines, trophoblastic primary cultures and third trimester placental explants [36,49,50]. InlB is also involved in the invasion of Jar cells and placental explants, in which *L. monocytogenes* ∆*inlA*, ∆*inlB,* and ∆*inlAB* mutants’ entry levels are identical [36,50]. In apparent contradiction with these *in vitro* and *ex vivo* results, InlA is not involved in placental infection in guinea pig, which is permissive to InlA, but not InlB [6,49]. In addition, InlB plays no role in placenta infection in wild type mice, which are permissive to InlB, but not InlA [6,51]. In contrast in gerbil and KIE16P mice, which are permissive to both InlA and InlB, these two factors are both involved in placenta and fetus invasion and mediate a massive fetal lethality after maternal oral inoculation [6]. These results indicated that the interactions of InlA and InlB with their respective receptor Ecad and c-Met act in a conjugated and interdependent manner to mediate placental invasion *in vivo* [6]. Indeed, although InlA mediates *L. monocytogenes* binding to Ecad-expressing cells, PI3-K activation is required for InlA-dependent internalization and InlB-c-Met interaction activates PI3-K [52,53]. In contrast to the intestinal epithelial cells targeted by *L. monocytogenes* [54], PI3-K is inactive at the basal state in SYN [50]. InlB interaction with c-Met is therefore critical for PI3-K activation in SYN and required for InlA-mediated *L. monocytogenes* breaching of the placental barrier [50] (Figure 1).

*Other bacterial factors involved in placental invasion –* Listeriolysin O (LLO) is a pore-forming toxin, which mediates *L. monocytogenes* escape from its internalization vacuole and modulates a large array of host signaling pathways [55]. As expected from its central role in virulence, LLO is involved in *L. monocytogenes* replication in mouse placenta, when a sufficient amount of *L. monocytogenes* is injected to force entry [51]. ActA mediates actin polymerization leading to the formation of comet tails which propel *L. monocytogenes* in the cytoplasm and intercellularly via protrusions. ActA is also involved in *L. monocytogenes* fetal-placental infection in mice as well as in guinea pigs [51,56]. This illustrates that LLO and ActA play major roles in *L. monocytogenes* intracellular survival, growth and propagation in the fetal-placental unit.

A screen by negative selection of hypovirulent mutants in an intravenously-inoculated guinea pig infection model led to the identification of an additional factor involved in placental colonization, which was called InlP [57]. The *inlP* gene productbelongs to the internalin family and interacts with afadin, a cytoplasmic protein associated with cell junctions [58]. Deletion of *inlP* also leads to a decrease of infection of the liver, spleen, and uterus of non-pregnant intravenously inoculated mice, indicating that its action is not specific to the placenta, consistent with afadin expression pattern [57].

The analysis of the pan-genome of the 6,600 strains of *L. monocytogenes* allowed highlighting specific hypervirulent CC-specific accessory genes, absent from the core genome of *L. monocytogenes*, which contains the aforementioned *L. monocytogenes* virulence factors [27]. A cluster of six genes annotated as encoding aputative sugar transporter system of the PTS family, named LIPI-4, is present in CC4, a CC significantly associated with MF listeriosis and neurolisteriosis in human. Deletion of LIPI-4 in CC4 results in a decrease of CNS infection in non-pregnant KIE16P mice, as well as a decrease in placental and fetal infection, but not of the maternal organs in pregnant KIE16P mice [27]. The mechanism by which LIPI-4 is involved in *L. monocytogenes* fetal-placental infection is unknown.

*Host immune response to L. monocytogenes infection* – Viviparity in mammals requires that maternal tissues tolerate the fetal allograft. At low dose, *L. monocytogenes* is contained by the colony stimulating factor CSF-1, which is secreted by the uterine epithelium and acts on trophoblasts that express CSF-1 receptor in experimentally infected mice, leading to the secretion of chemoattractants of inflammation proteins (MIP)-2 by trophoblasts, that recruit neutrophils and macrophages in the decidual tissue to control infection [59,60]. In human, *L. monocytogenes* infection mainly leads to fetal loss by spontaneous abortion [5]. In mice, Foxp3^+^ regulatory T cells (T-regs), which are involved in immune tolerance toward the fetus enhance susceptibility to *L. monocytogenes* infection [61]. During pregnancy, *L. monocytogenes* induces fetal injury and eventually fetal resorption, directly by infection of the fetus, or indirectly by inhibiting the fetal tolerance property of T-regs [62]. Fetal resorption itself is induced by the influx of inflammatory neutrophils and macrophages, leading to the recruitment of maternal T cells specific to fetal antigens. These CD8^+^ T cells activate the expression of the chemoreceptor CXCR3, leading to the accumulation of CD8^+^ T cells in the decidual tissue and fetal loss [63].

## Conclusions

Listeriosis is a rare but extremely severe foodborne maternal-neonatal infection, that frequently leads to fetal loss and neonatal infection. *L. monocytogenes* has proven as an invaluable model microorganism to decipher the events associated with placental invasion by a pathogen; in the same way, it has allowed key discoveries in basic immunology and cell biology. MN listeriosis is a direct consequence of *L. monocytogenes* specific placental tropism, which is mediated by the conjugated action of InlA and InlB at the placental barrier. Other key virulence factors of *L. monocytogenes*, such as ActA and LLO, are also involved in *L. monocytogenes* replication in the placental and dissemination into fetal tissues, in a nonspecific manner, as is InlP. LIPI-4 is also involved in placental infection, as well as CNS infection, by so far unknown mechanisms. Despite these advances, a number of scientific, experimental, and medical challenges remain to fully understand how and why *L. monocytogenes* is able to reach and invade so efficiently and silently the placenta and induce its devastating consequences on the developing fetus.
